# AI Software Among Commercially Insured Populations: Cross-Sectional Study of Patient and Plan Characteristics

**DOI:** 10.2196/92726

**Published:** 2026-07-23

**Authors:** Elsa Zhang, Yujia Jin, Ching-Ching Claire Lin, Raymond Kuo, Joshua M Liao

**Affiliations:** 1 Program on Policy Evaluation and Learning, Dallas, TX, United States; 2 Department of Internal Medicine, Division of General Internal Medicine, The University of Texas Southwestern Medical Center, 5323 Harry Hines Blvd, Dallas, TX, United States, 1 214 648 3111; 3 Institute of Health Policy and Management, College of Public Health, National Taiwan University, Taipei, Taiwan; 4 Global Health Program, College of Public Health, National Taiwan University, Taipei City, Taiwan; 5 Population Health Research Center, National Taiwan University, Taipei City, Taiwan

**Keywords:** software-as-a-service, radiology, health policy, Medicare, digital innovation

## Abstract

We describe the adoption of AI software among commercially insured adults from 2018 to 2023 and find rapid growth but variable use across patients, plans, and regional characteristics, highlighting the need for research and policy to support equitable and beneficial adoption.

## Introduction

AI is poised to transform radiology and imaging workflows [1-3]. Despite this potential, prior work has often focused on Medicare populations [4,5]. Less is known about how AI has been used among commercially insured patients, with only one other analysis addressing this topic to our knowledge and leaving two major knowledge gaps: insight about use across patient characteristics and insurance plans [6].

## Methods

### Study Design

We used national claims data from Merative MarketScan to identify reimbursed AI software services using Current Procedural Terminology (CPT) codes: CPT 0501T-0504T (AI-enabled Fractional Flow Reserve Derived From Computed Tomography); CPT 0615T (AI-enabled Eye-Movement Analysis Without Spatial Calibration); CPT 92229 (AI-enabled Imaging of Retina for Detection or Monitoring of Disease); CPT 0623T-0626T (AI-enabled Atherosclerosis Imaging-Quantitative Computer Tomography); CPT 0648T-0649T (AI-enabled LiverMultiScan Service); CPT 0697T-0698T (AI-enabled Quantitative Magnetic Resonance for Analysis of Tissue Composition); CPT 0721T-0722T (AI-enabled Optellum Lung Cancer Prediction); CPT 0723T-0724T (AI-enabled Quantitative Magnetic Resonance Cholangiopancreatography); CPT 0764T-0765T (AI-enabled Low Ejection Fraction AI-ECG Service); CPT 0808T (AI-enabled XV Lung Ventilation Analysis Software); and CPT C9786 (AI-enabled EchoGo Echocardiography Image Processing Service). Codes were included to reflect reimbursable AI software identified through a literature review of Medicare rulings and Medicare Payment Advisory Commission reports using “artificial intelligence” and “software-as-a-service” terms [7,8].

We included beneficiaries aged 18 years or older who used AI software between 2018 and 2023 and had at least 11 of 12 months of continuous enrollment in the year preceding AI software use. Patient characteristics included age, sex, geographic region, clinical complexity (Charlson Comorbidity Index; CCI), urban residence (Metropolitan Statistical Area), and insurance plan type (health maintenance organization, preferred provider organization, high-deductible, other). Analyses were conducted in Snowflake SQL (version 9.23.1) and Python (version 3.13.2; Anaconda Inc).

### Ethical Considerations

This study was considered non–human subjects research and exempted from institutional review board per institutional policy under 45 CFR 46.102.

## Results

**Figure 1.**
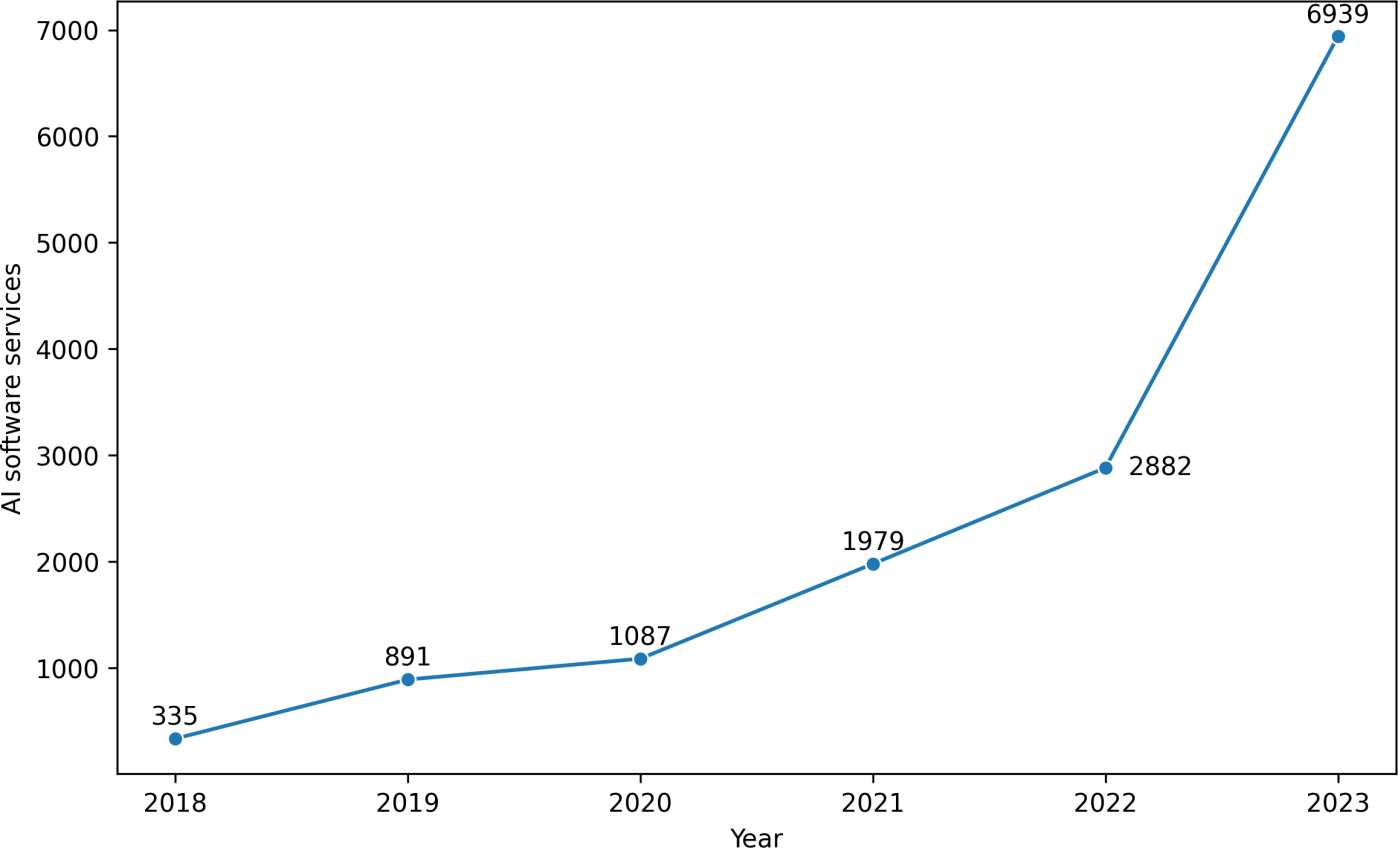
Adoption of AI software among commercially insured patients in the United States.

AI software was used a total of 14,133 times in the care of 8272 of a total of 15,994,063 eligible patients. Use increased by nearly 2000% from 335 services in 2018 to 6939 services in 2023. Fractional flow reserve derived from computed tomography was used most frequently, representing 74.93% (n=10,575) of all services over the study period (Figure 1, Multimedia Appendix 1).

AI software was used more frequently among male (n=5140, 62.14%) than female patients (n=3132, 37.86%) and more frequently in metropolitan areas (n=6318, 89.04%) than micropolitan or nonurban areas. The mean age of patients using AI software was 53.95 years old (SD 9.02), and the mean CCI of patients was 1.45 (SD 1.86). Use was highest in the South (n=3641, 44.02%), followed by the North Central (n=2424, 29.30%), West (n=1160, 14.02%), and Northeast (n=1040, 12.57%) (Table 1) regions.

AI software use was more common among preferred provider organization (n=3585, 44.30%) and high-deductible (n=2588, 31.98%) insurance plans, and far less frequent among health maintenance organization (n=1008, 12.46%) and other (n=911, 11.26%) plans (Table 1).

**Table 1.** Characteristics of commercially insured patients in the United States with AI software use.

Characteristic	Value
Age (years)^a^, mean (SD)	53.95 (9.02)
Sex^a^, n (%)	
Male	5140 (62.14)
Female	3132 (37.86)
CCI^b^, mean (SD)	1.45 (1.86)
Geographic region^c^, n (%)	
South	3641 (44.02)
North Central	2424 (29.30)
Northeast	1040 (12.57)
West	1160 (14.02)
Unknown	7 (0.08)
Residence in metropolitan area^a^, n (%)	6318 (89.04)
Plan type^d^, n (%)	
PPO^e^	3585 (44.30)
HMO^f^	1008 (12.46)
High-deductible	2588 (31.98)
Other	911 (11.26)

a N=8272.

b n=8228.

c n=7096.

d n=8092.

e PPO: preferred provider organization.

f HMO: health maintenance organization.

## Discussion

Among commercially insured patients, AI software use has increased rapidly. This trend may reflect early diffusion alongside expanding US Food and Drug Administration–cleared AI applications and evolving reimbursement and coding pathways, although adoption remained disproportionately concentrated within a limited number of services [4-7,9]. Future work should evaluate how other factors, such as market changes, may also affect use. While exploratory and descriptive, these results point to several key directions for future work as AI adoption increases more broadly.

Future research can identify factors driving variation in AI software use across patients, insurance plan types, and regions. Understanding facilitators and barriers is critical, as AI may exacerbate existing health disparities, particularly considering the longstanding inequities in access to imaging and emerging technologies among underserved populations [10,11]. Given documented variation in structures and strategies between different insurance plans, the variation across insurance plan types observed in this analysis suggests that the adoption and diffusion of AI software may differ by plan structure. Greater use may be more prevalent among more flexible (eg, network breadth) and costly (eg, higher co-pays and deductibles) insurance plans, but less prevalent among plans characterized by tighter networks, primary-care gatekeeping, and robust utilization management, such as health maintenance organizations [12]. The variation across geography observed in this analysis may be in part explained by the presence of well-resourced health care delivery organizations that are able to make technology and organizational infrastructure investments, as well higher disease prevalence of diseases aligned with AI software [13,14].

Our findings are consistent with prior studies on AI adoption. Analyses of Medicare patients found that adoption of one single AI service was more prevalent among hospitals and clinicians with greater resources, and among radiologists, both adoption and denial rates were notable [4-6]. Analyses among commercial populations found that utilization was concentrated within a limited number of AI services with geographic variation, but these did not examine patient characteristics [6]. No prior research has evaluated variation by insurance plan type.

While future work is needed to assess the dynamics, our study fills gaps in the relatively nascent area of AI software adoption among commercial patients, examining differences in AI software adoption across insurance plan types and patient characteristics. These findings provide foundational data for future analytic studies evaluating determinants of adoption and guiding policy design.

Study limitations include exploratory intent, descriptive design, and small sample size, which preclude causal inferential analysis about associations between AI software use and outcomes, which should be the focus of future research. Additionally, this analysis may not capture all types of AI services used clinically. Future studies should include other types of AI services, such as those deployed within hardware (software in a medical device) and those delivered through local access (eg, installation within local systems or via electronic health records).

Nonetheless, by describing characteristics of commercially insured patients and plans in which AI software is used, this study underscores the need for future research and policy to ensure equitable and beneficial diffusion of AI software.

## Supplementary material

10.2196/92726 Multimedia Appendix 1 AI software adoption by service.

## Abbreviations:

CCI: Charlson Comorbidity Index
CPT: Current Procedural Terminology
