# Supplementary material for: AI Software Among Commercially Insured Populations: Cross-Sectional Study of Patient and Plan Characteristics
Source: JMIR Form Res. 2026 Jul 23;10:e92726. doi: 10.2196/92726 (PMC13394851; doi:10.2196/92726)
Supplement: Multimedia Appendix 1 [file formative-v10-e92726-s001.docx]

**Table 1.** AI Software Adoption by Service

| AI Software | Services |
| --- | --- |
| AI-enabled Fractional Flow Reserve Derived From Computed Tomography  (CPT 0501T–0504T ) | 10575 |
| AI-enabled Eye-Movement Analysis Without Spatial Calibration  (CPT 0615T) | 15 |
| AI-enabled Imaging of Retina for Detection or Monitoring of Disease  (CPT 92229) | 1535 |
| AI-enabled Atherosclerosis Imaging-Quantitative Computer Tomography  (CPT 0623T–0626T) | 256 |
| AI-enabled LiverMultiScan Service  (CPT 0648T–0649T) | 158 |
| AI-enabled Quantitative Magnetic Resonance for Analysis of Tissue Composition  (CPT 0697T–0698T) | 44 |
| AI-enabled Optellum Lung Cancer Prediction  (CPT 0721T–0722T) | 0 |
| AI-enabled Quantitative Magnetic Resonance Cholangiopancreatography  (CPT 0723T–0724T) | 104 |
| AI-enabled Low Ejection Fraction AI-ECG Service  (CPT 0764T–0765T) | 1426 |
| AI-enabled XV Lung Ventilation Analysis Software  (CPT 0808T) | 0 |
| AI-enabled EchoGo Echocardiography Image Processing Service  (CPT C9786) | 0 |
| Total Services | 14113 |

**Table 2.** AI Software Adoption Frequency

| Services | Patients |
| --- | --- |
| 1 | 4637 |
| 2 | 1556 |
| 3 | 2014 |
| 4 | 36 |
| 5 | 11 |
| 6 | 13 |
| 7 | 2 |
| 8 | 2 |
| 15 | 1 |
| Total Services | 14113 |
| Total Patients | 8272 |


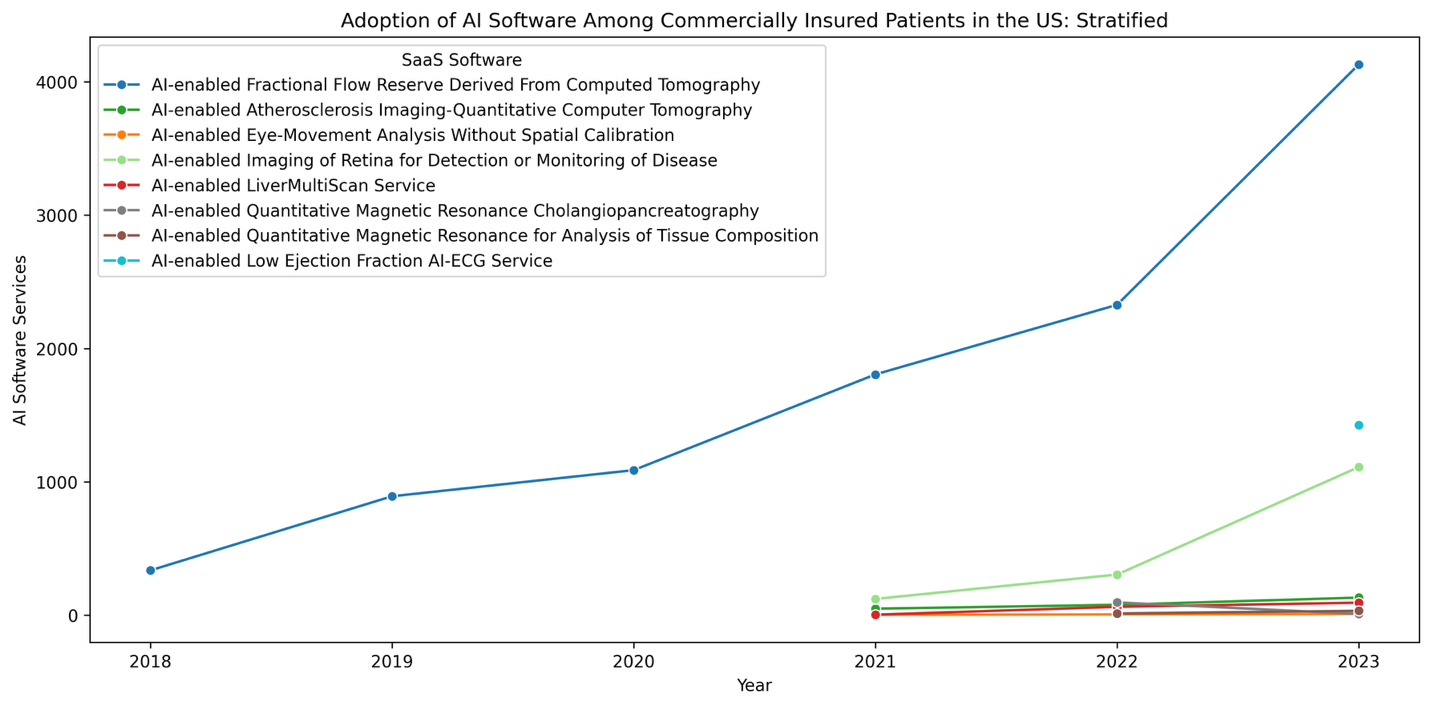


**Figure**. Adoption of AI Software Among Commercially Insured Patients in the U.S: Stratified
